# Serum albumin and blood urea as independent predictors of in-hospital mortality in hospitalized COVID-19 patients: A retrospective cohort study

**DOI:** 10.1371/journal.pone.0353456

**Published:** 2026-07-08

**Authors:** Nuha Al-Aghbari

**Affiliations:** 1 Department of Community Medicine, Faculty of Medicine and Health Sciences, University of Science and Technology, Sana’a, Yemen; 2 Faculty of Medicine, Doctoral Program of Public Health, Universitas Sebelas Maret, Surakarta, Indonesia; Sai Gosavi Specialty Clinic / Nano Hospitals Bangalore / Saraswati Specialty Clinic, INDIA

## Abstract

**Background:**

While systemic inflammation is a hallmark of severe COVID-19, the prognostic value of metabolic and organ-functional markers remains under-explored. This study examined the prognostic value of serum albumin and blood urea at hospital admission in predicting in-hospital mortality among patients with COVID-19.

**Methods:**

We performed a retrospective cohort study of 1,074 adult patients with laboratory-confirmed COVID-19 who were hospitalized in a tertiary care center. Patients’ demographic, clinical and laboratory data on admission were collected from electronic medical records. We conducted multivariable logistic regression analyses to determine independent predictors of in-hospital death after adjusting for possible confounders including age, sex, comorbidities and known inflammatory/coagulation markers. Missing laboratory variables were imputed by multiple imputation by chained equations. Area under the receiver Operating characteristics curve (AUC) was evaluated for model discrimination.

**Results:**

In-hospital mortality occurred in (24.5%). Non-survivors had significantly lower serum albumin and higher blood urea levels at admission. After full adjustment, hypoalbuminemia remained independently associated with increased mortality risk. Each 10 mg/dL increase in urea was associated with a 14% increase in the odds of death (AOR = 1.14, 95% CI 1.08–1.20), whereas albumin demonstrated an independent protective association with mortality (AOR = 0.52, 95% CI 0.37–0.74). The final multivariable model demonstrated good discrimination (AUC = 0.86), indicating strong predictive ability.

**Conclusions:**

Serum albumin and blood urea at hospital admission independently predict mortality in patients with COVID-19, even after accounting for inflammatory and coagulation markers. These findings suggest that markers of organ-functional reserve may provide prognostic information beyond traditional inflammatory markers in hospitalized patients with COVID-19.

## Introduction

The severe acute respiratory syndrome coronavirus 2 (SARS-CoV-2), which causes coronavirus disease 2019 (COVID-19), has been associated with substantial global morbidity and mortality, particularly among hospitalized patients with severe disease. While most infected individuals experience mild-to-moderate symptoms, a subset develop critical complications including acute respiratory failure, multi-organ dysfunction, and systemic inflammatory response syndrome [1,2]. Early identification of patients at high risk of mortality remains essential for optimizing clinical management and resource allocation [3].

Advanced age, male sex, and pre-existing comorbidities have consistently been associated with increased mortality risk in hospitalized patients with COVID-19 [4,5]. In addition, inflammatory and immune-related biomarkers, including C-reactive protein (CRP), lactate dehydrogenase (LDH), D-dimer, and neutrophil-to-lymphocyte ratio (NLR), have demonstrated prognostic utility [6]. However, comparatively less attention has been given to markers reflecting hepatic synthetic function and renal metabolic stress, despite the multisystem nature of severe COVID-19 illness.

Hepatic dysfunction in COVID-19 may result from inflammatory injury, hypoxia, microvascular thrombosis, and direct viral effects [7]. Serum albumin reflects hepatic synthetic capacity, systemic inflammation, endothelial integrity, and nutritional status [8]. Hypoalbuminemia has consistently been associated with poor outcomes in critically ill patients, including those with COVID-19 [9,10]. Similarly, renal involvement in severe COVID-19 has been associated with acute kidney injury and increased mortality [10,11]. Elevated blood urea may reflect renal hypoperfusion, metabolic stress, and systemic catabolism during severe illness [12]. Although hepatic and renal abnormalities have individually been associated with adverse outcomes, their combined prognostic relevance has not been sufficiently evaluated.

Thus, the present study sought to examine the independent and combined associations of serum albumin and blood urea levels with in-hospital mortality among hospitalized patients with laboratory-confirmed COVID-19. The study also evaluated whether these associations remained significant after adjustment for demographic factors, co-morbidities, and established inflammatory markers.

## Materials and methods

### Study design, population, and setting

Based on guidelines for cohort studies provided by Strengthening the Reporting of Observational Studies in Epidemiology (STROBE), this study has been reported [See Supporting Information S1 Table].

**Fig 1:**
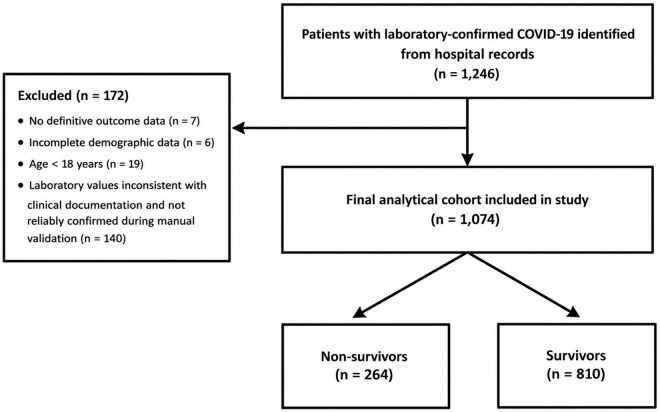
Flow diagram of patient selection and final analytical cohort.

A retrospective observational analysis of adult patients hospitalized with laboratory-confirmed COVID-19 was undertaken from October 2020 to September 2021 in a tertiary care facility, in Belgaum, Karnataka, India. The study sample included all eligible hospitalized patients during the study period. We initially identified 1,246 patients with laboratory-confirmed COVID-19 (RT-PCR or rapid antigen test). Electronic medical records were accessed for research purposes between [10/ 10/ 2021] and [25/ 11/ 2021]. After a rigorous manual review of the electronic medical records, we excluded 172 cases to maintain data quality. Seven patients lacked definitive outcome data, 6 had incomplete demographics, and 19 were under 18 years of age. Laboratory values were screened using predefined plausibility ranges derived from reference standards. Values outside physiologically possible ranges or discordant with contemporaneous clinical parameters were verified manually. Records containing laboratory values that were inconsistent with accompanying clinical documentation and could not be reliably confirmed during data validation. These records (n = 140) were excluded prior to multiple imputation to maintain data quality. To assess potential selection bias, demographic characteristics and mortality rates were compared between excluded and included patients. No clinically meaningful differences were identified [See Supporting Information S2 Table]. Our final analytical dataset consisted of 1,074 patients as shown in Fig 1.

### Data collection

Relevant characteristics were extracted from hospital patients’ medical records using a standardized data collection form. Under the guidance of the lead investigator (N.A.), two skilled research assistants manually retrieved data from hospital charts; a clinical consultant subsequently validated the acquired data. In order to guarantee correctness and consistency, a one-week training session on data extraction methods, coding manual usage, and verification standards was conducted prior to data collection.

Ten percent of the records were randomly rechecked for quality and completeness, and data collectors used a standardized, pre-tested data extraction form. The study team discussed and worked out any disagreements.

The study variables were recorded using a pre-made data-collection sheet. In-hospital mortality among patients with a confirmed COVID-19 infection, categorized as survivors or non-survivors, was the main outcome variable.

Clinical and laboratory data were collected at the time of hospital admission. We focused on standard metabolic and inflammatory markers to evaluate multi-system involvement. Serum albumin and blood urea were selected as markers representing hepatic synthetic function and renal metabolic stress, respectively. Other covariates included age, sex, pre-existing comorbidities, and inflammatory indices such as C-reactive protein (CRP), Lactate Dehydrogenase (LDH), and the Neutrophil-to-Lymphocyte Ratio (NLR).

### Statistical strategy

Stata MP 64-bit, version 17.0 (Stata Corp LLC, College Station, TX, USA) was used for data analysis. Given the non-normal distribution of our continuous variables, we reported data as Medians and Interquartile Ranges (IQR) and utilized the Mann-Whitney U test for group comparisons [Provided in Supporting Information S3 Table]. Categorical data were compared using Chi-square tests. The proportion of missing laboratory data ranged from 9.7% (hemoglobin) to 33.9% (prothrombin time). Key variables including LDH (32.8%), albumin (28.2%), urea (25.0%), and D-dimer (26.3%) had substantial missingness. Patterns of missingness were examined and were consistent with a missing at random assumption. To minimize bias associated with complete-case analysis, Multiple Imputation by Chained Equations (MICE) was performed. 30 imputed datasets were generated. Continuous variables were imputed using predictive mean matching, binary variables using logistic regression, and skewed biomarkers were log-transformed prior to imputation. Incorporating all predictors and the mortality outcome variable. Estimates from imputed datasets were combined using Rubin’s rules. To assess the plausibility of the Missing at Random (MAR) assumption, we examined associations between missingness of key laboratory variables (albumin, urea, LDH, and D-dimer) and baseline characteristics, including age, sex, comorbidities, and mortality status, using logistic regression models. Mortality was not a significant predictor of missing laboratory values. Baseline characteristics were also compared between complete and incomplete cases. As a sensitivity analysis, the primary multivariable model was re-estimated using complete-case data only; the direction and magnitude of associations were consistent with those observed in the imputed analysis. [See Supporting Information S4 Table].

This approach preserved statistical power for multivariable modeling. Variables with substantial missingness were retained in the primary model because of their established clinical relevance in COVID-19 prognosis and the consistency of findings observed in sensitivity analyses. Variable selection followed a structured two-step process. First, crude odds ratios (ORs) were estimated for all baseline variables in univariable analyses. Selection for multivariable modeling was guided by both clinical relevance and statistical considerations. Variables demonstrating a univariable association with mortality at p < 0.20 were considered for inclusion to reduce the risk of excluding potentially important confounders. This liberal screening threshold is supported by established epidemiological modeling guidance, which cautions against overly restrictive criteria at the univariable stage [13]. Clinically important variables identified a priori from prior literature were retained regardless of statistical significance. Selected variables were entered simultaneously into the final multivariable logistic regression model to estimate adjusted odds ratios (AORs).

A supplementary sensitivity analysis was performed in the subgroup of patients with available serum creatinine measurements to evaluate whether the observed association between urea and mortality remained significant after adjustment for creatinine [See supporting information S9 Table].

Model discrimination was assessed using the area under the receiver operating characteristic curve (AUC). Multicollinearity was evaluated using variance inflation factors (VIF), with all VIF values < 5, indicating no evidence of significant multicollinearity [See Supporting Information S5 Table]. Model calibration was assessed using the Hosmer–Lemeshow goodness-of-fit test **[**Results Attached in Supporting Information S6 and S7 Tables].

To evaluate potential effect modification between hepatic and renal functional markers, an interaction term (albumin × urea) was included in the multivariable model. The interaction was not statistically significant, and detailed results are provided in the Supporting File S8 Table.

### Ethic statement

The Institutional Human Ethics Committee of Jawaharlal Nehru Medical College, KLE Academy of Higher Education and Research, Belagavi, Karnataka, India, granted ethical clearance for this study [Ref. No.: MDC/DOME/243, dated 5 October 2021]. The requirement for informed consent was waived due to the retrospective use of anonymized data. The committee determined that the study protocol was both justified and ethically acceptable. The Clinical Services Administrator, Medical Director, and Chief Executive Officer of KLE Hospital, as well as the Medical Records Department, granted permission for the access and use of de-identified patient records. During the data extraction process, investigators had temporary access to hospital identification numbers solely for the purpose of record verification; however, no directly identifiable personal information (such as names or contact details) was retained in the analytical dataset. The research was conducted in accordance with the institutional guidelines and the ethical principles outlined in the Declaration of Helsinki. In order to protect participant confidentiality, the data were completely anonymized and the most recognizable identifiers were eliminated during analysis.

## Results

### Baseline characteristics

Table 1 shows the baseline characteristic of the 1,074 COVID-19 patients were included in the study, categorized into survivors and non-survivors. The median age of the entire cohort was 54 years (IQR: 25), with non-survivors being significantly older than survivors (59 vs. 51 years; p < 0.001). Comorbidities were significantly more prevalent in the non-survivor group (48.4% vs. 36.0%; p < 0.001). The primary hepato-renal functional markers demonstrated marked differences at baseline.

**Table 1:** Baseline Demographics and Clinical Characteristics of Survivors vs. Non-Survivors.

Variable	Total (N = 1,074)	Survivors (n = 810)	Non-Survivors (n = 264)	p.value
**Age (years)**	54 (25)	51 (26)	59 (22)	< 0.001
**Sex**				
Male	723 (67.3)	537 (66.3)	186 (70.4)	**0.211**
Females	351 (32.6)	273 (33.7)	78 (29.5)	
**Comorbidities**				
Yes	420 (39.1)	292 (36.0)	128 (48.4)	**<0.001**
No	654 (60.8)	518 (63.9)	136 (51.5)	
**Hb (**g/dL)	12.8 (2.7)	12.9 (2.6)	12.7 (3.2)	0.049
**WBC (**×10³/µL)	9.3 (7.1)	8.6 (5.8)	12.51 (9.4)	**<0.001**
**Platelets (**×10³/µL)	203 (112)	207 (111)	191 (119.8)	**0.008**
**NLR**	6.6 (11.1)	5.4 (7.8)	14.6 (16.6)	**<0.001**
**LDH (per 100 U/L)**	407.8 (347)	359.5 (273)	628.5 (400.5)	**<0.001**
**hs-CRP (**mg/L)	75.7 (133.8)	62.25 (109.8)	132.9 (155.4)	**<0.001**
**D.dimer (**ng/mL)	682.5 (1,134)	575 (1028)	1150.5 (4,372)	**<0.001**
**Urea (per 10 mg/dL)**	36 (39)	30 (28)	60.18 (53.5)	**<0.001**
**Albumin (g/dL)**	3.5 (0.7)	3.6 (0.8)	3.2 (0.6)	0.001
**PT (**seconds)	13.8 (4.5)	13.5 (4.1)	14.8 (5.1)	**<0.001**
**AST (**U/L)	40 (54)	38 (52)	47.21 (52.4)	**0.001**
**Na (**mmol/L)	136 (7)	136 (7.0)	138 (8)	< 0.001
**K (**mmol/L)	4.1(0.9)	4.1 (0.8)	4.3 (1.1)	0.001
**HCO3 (**mmol/L)	20 (8)	20 (7)	19 (9.7)	**0.007**

Continuous variables are expressed as Median (Interquartile Range). Categorical variables are expressed as frequency (percentage). Interquartile Range (IQR) represents the range from the 25th to the 75th percentile. P-values were calculated using the Mann–Whitney U test for continuous variables and the Chi-square test for categorical variables.

Non-survivors exhibited significantly higher median Urea levels (60.18 mg/dL vs. 30 mg/dL; p < 0.001) and significantly lower median Albumin levels (3.2 g/dL vs. 3.6 g/dL; p = 0.001) compared to survivors. Inflammatory and coagulation markers, including WBC, NLR, LDH, CRP, and D-dimer, were also significantly elevated among non-survivors’ patients (all p < 0.001).

### Bivariate and multivariable predictors of mortality

In the univariable logistic regression analysis, almost all clinical and laboratory parameters were associated with mortality. Notably, Albumin showed a strong protective effect with a crude odds ratio (OR) of 0.29 (95% CI: 0.22–0.38; p < 0.001), while Urea was associated with increased mortality risk (OR: 1.24; 95% CI: 1.19–1.30; p < 0.001) as shown in Table 2.

**Table 2:** Univariable Logistic Regression Analysis for Factors Associated with COVID-19 Mortality.

Variable	Crude OR	95%CI	p.value
**Age**	1.03	1.02-1.04	**<0.001**
**Sex [ref: Male]**			
Females	0.82	0.60-1.11	0.211
**Comorbidities [ref: No]**			
Yes	1.66	1.26-2.21	**<0.001**
**Hb (**g/dL)	0.93	0.88-0.99	**0.050**
**WBC (**×10³/µL)	1.10	1.07-1.13	**<0.001**
**Platelets (**×10³/µL)	0.99	0.99-0.99	**0.016**
**NLR**	1.08	1.06-1.09	**<0.001**
**LDH (per 100 U/L)**	1.26	1.20-1.32	**<0.001**
**hs-CRP (**mg/L)	1.00	1.00-1.01	**<0.001**
**D.dimer (**ng/mL)	1.00	1.00-1.01	**<0.001**
**Urea (per 10 mg/dL)**	1.24	1.19-1.30	**<0.001**
**Albumin (g/dL)**	0.29	0.22-0.38	**<0.001**
**PT (**seconds)	1.03	1.01-1.06	**0.001**
**AST (**U/L)	1.00	0.99-1.00	0.558
**Na (**mmol/L)	1.05	1.03-1.07	**<0.001**
**K (**mmol/L)	1.42	1.19-1.68	**<0.001**
**HCO3 (**mmol/L)	0.98	0.96-0.99	**0.024**

Table 3 presents the multivariable logistic regression model. After adjusting for age, sex, comorbidities, and inflammatory markers, albumin and urea remained independently associated with mortality after multivariable adjustment. Albumin maintained a significant independent protective effect with an adjusted odds ratio (AOR) of 0.52 (95% CI: 0.37–0.74; p < 0.001), suggesting that for every 1 g/dL increase in albumin, the risk of death decreases by approximately 48%. Because relatively large changes in serum albumin are uncommon in acute clinical settings, the observed association should be interpreted as reflecting cumulative physiological vulnerability rather than abrupt threshold-based risk stratification.

**Table 3:** Multivariable Logistic Regression Analysis of Independent Predictors of Mortality.

Variable	AOR	95%CI	p.value
**Age**	1.02	1.01-1.03	**<0.001**
**Sex [ref: Male]**			
Females	1.36	0.90-2.04	0.138
**Comorbidities [ref: No]**			
Yes	1.17	0.80-1.71	0.395
**Hb (**g/dL)	1.06	0.97-1.17	0.158
**WBC (**×10³/µL)	1.04	1.01-1.07	**0.007**
**Platelets (**×10³/µL)	0.99	0.99-1.00	0.107
**NLR**	1.03	1.01-1.05	**0.001**
**LDH (per 100 U/L).**	1.17	1.11-1.23	**<0.001**
**hs-CRP (**mg/L)	1.003	1.001-1.005	**<0.001**
**D.dimer (**ng/mL)	1.01	1.00-1.01	**0.036**
**Urea (per 10 mg/dL)**	1.14	1.08-1.20	**<0.001**
**Albumin (g/dL)**	0.52	0.37-0.74	**<0.001**
**PT (**seconds)	1.02	1.00-1.04	**0.037**
**Na (**mmol/L)	1.02	1.00-1.05	**0.042**
**K (**mmol/L)	0.91	0.71-1.15	0.433
**HCO3 (**mmol/L)	1.003	0.985-1.021	0.731

Continuous laboratory variables were rescaled to clinically meaningful increments (urea per 10 mg/dL and LDH per 100 U/L). Odds ratios reflect these increments.

Urea remained an independent predictor of mortality (AOR: 1.14 per 10 mg/dL increase; 95% CI: 1.08–1.20; p < 0.001). When scaled to clinically relevant increments, this corresponds to approximately a 14% increase in mortality odds per 10 mg/dL increase and a 35% increase per 30 mg/dL increase.

Other independent predictors included Age (AOR: 1.02), WBC (AOR: 1.04), NLR (AOR: 1.03), and LDH (AOR: 1.00) (all p < 0.05).

Complete-case analysis including albumin, urea, age, sex, and comorbidity (n = 755) yielded effect estimates consistent in direction and magnitude with the primary imputed model (Albumin OR 0.41; Urea per 10 mg/dL OR 1.20), supporting the robustness of the findings (results attached in supplementary file).

A supplementary sensitivity analysis was additionally performed in the subset of patients with available serum creatinine measurements (n = 853). After adjustment for serum creatinine and other covariates, blood urea remained independently associated with mortality (AOR = 1.13, 95% CI: 1.06–1.21; p < 0.001), whereas creatinine itself was not independently associated with mortality (AOR = 0.94, 95% CI: 0.82–1.09; p = 0.445). [See supporting information S9 Table].

The interaction term between albumin and urea was not statistically significant (OR 1.0009, 95% CI 0.993–1.008; p = 0.817), indicating no evidence of a multiplicative interaction between the two variables. Accordingly, the final model retained only the main effects. Results provided in supplementary file.

The Hosmer–Lemeshow goodness-of-fit test indicated adequate model calibration (χ² = 7.15, p = 0.52).

**Fig 2:**
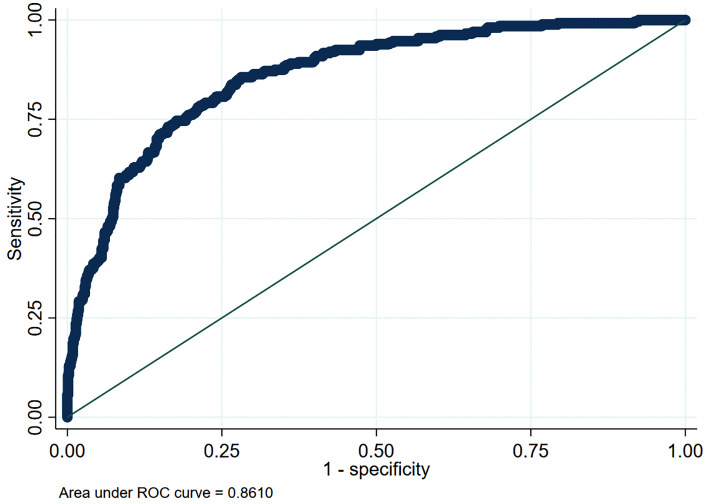
ROC Curve Analysis for the Prediction of In-Hospital Mortality.

The diagnostic performance of the multivariable prognostic model was assessed using Receiver Operating Characteristic (ROC) curve analysis Fig 2. The model demonstrated good discriminatory capacity for in-hospital mortality, with an Area Under the Curve (AUC) of 0.8609. This indicates that, in randomly selected pairs of survivors and non-survivors, the model correctly assigns a higher predicted probability of death to the non-survivor approximately 86% of the time. The ROC curve’s proximity to the upper-left corner reflects strong discriminatory ability across a range of classification thresholds, supporting the model’s potential utility for clinical risk stratification.

The optimal probability threshold identified using the Youden index was 0.35, yielding a sensitivity of 82% and specificity of 78%.

**Fig 3:**
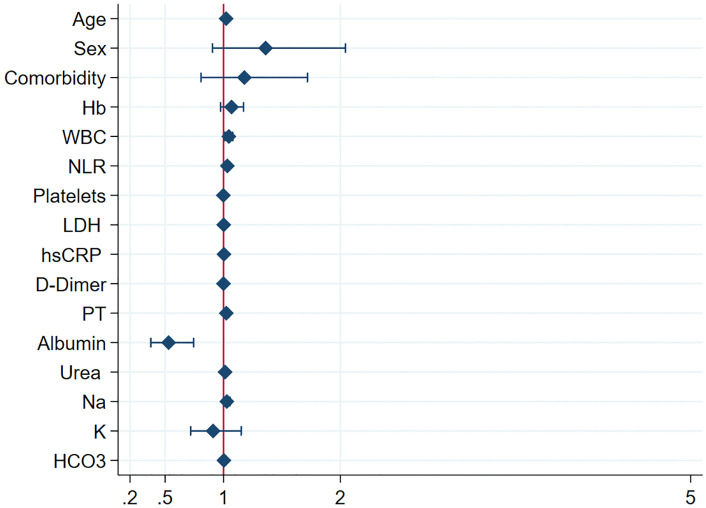
Forest Plot of Adjusted Odds Ratios for Mortality Predictors.

Fig 3 presents the forest plot that represents the independent impact of clinical and biochemical variables on mortality risk, as determined by multivariable logistic regression. Albumin emerged as the primary independent protective factor. With an adjusted odds ratio (AOR) of 0.52 (95% CI: 0.37–0.74), its position far to the left of the unity line (1.0) visually confirms that higher albumin levels are associated with a nearly 48% reduction in the odds of mortality.

Age (AOR 1.02) and Urea (AOR 1.14) were confirmed as independent risk factors. Several baseline parameters, including Sex, Comorbidities, and Hemoglobin (Hb), had confidence intervals that crossed the 1.0 threshold, indicating that they did not maintain independent prognostic significance after adjusting for albumin, urea, and other inflammatory markers

## Discussion

This study used a retrospective cohort that included 1,074 COVID-19 patients. While inflammatory biomarkers such as CRP, LDH, and D-dimer have dominated prognostic models during the pandemic, our findings suggest that organ-functional markers may reflect a later and more clinically decisive stage of systemic decompensation. Severe SARS-CoV-2 infection is increasingly recognized as a syndrome of endothelial injury, microvascular thrombosis, and dysregulated host response rather than isolated pulmonary pathology.

Although approximately 25–33% of the selected laboratory variables had missing values, sensitivity analyses restricted to complete cases yielded similar effect estimates, suggesting that the imputation procedure did not materially influence the observed associations.

The hypoalbuminemia in this context is most likely the result of a combination of processes: decreased hepatic synthetic function due to cytokine-mediated inhibition, increased capillary permeability leading to transcapillary albumin loss, and increased catabolic rates [14]. Albumin is a negative acute phase protein, and its decrease is proportional to the activation of systemic inflammation; it is, however, the only protein that reflects nutritional reserve, oncotic stability, and hepatic function [15]. This complex role may account for the fact that albumin was independently protective even after adjusting for CRP and LDH.

Furthermore, albumin contributes to antioxidant buffering and endothelial stabilization. Reduced levels may exacerbate oxidative stress and microvascular injury, both of which are central to severe COVID-19 pathogenesis [16]. Thus, hypoalbuminemia is not merely a passive marker but may reflect a biologically vulnerable state.

After adjustment for multiple variables, the association of albumin with mortality remained statistically significant, but the absolute difference in median albumin levels between survivors and non-survivors was relatively small (3.6 vs 3.2 g/dL). Both values are within a range that is usually clinically interpreted as mild to moderate hypoalbuminemia. Therefore, albumin should not be interpreted as an isolated diagnostic threshold for mortality risk, but rather as a marker of broader systemic physiological deterioration. The prognostic value of albumin is more likely to be informative when interpreted with other inflammatory and organ-functional markers.

### Renal metabolic stress as a mortality signal

Elevations in blood urea levels during critical illness may reflect renal hypoperfusion, neurohormonal activation, systemic catabolism, and hemodynamic instability.

SARS-CoV-2 infection induces endothelial dysfunction through direct viral invasion, inflammatory cytokine activation, and microvascular injury, which leads to thrombotic microangiopathy that makes kidneys more vulnerable to damage. Reports published in Kidney International have shown that hospitalized patients develop acute kidney injury at high rates [1,17], supporting the prognostic importance of renal involvement in severe COVID-19.

Yin et al. [18] demonstrate that urea serves as a better early indicator of systemwide body decline because it reacts to changes in body fluid levels and protein breakdown activities. Our findings demonstrate a graded association between increasing urea levels and mortality risk, even at relatively small increments. The effect size becomes substantial when we scale it to measures that clinician commonly encounter in practice which may improve understanding of its clinical implications.

Interestingly, in a supplementary analysis including serum creatinine, the association between urea and mortality remained significant whereas creatinine was not independently associated with mortality. This finding suggests that urea may capture broader physiological disturbances beyond glomerular filtration alone, including systemic catabolic stress, neurohormonal activation, and hemodynamic instability during severe illness.

Consistent with prior studies demonstrating strong associations between acute kidney injury, elevated blood urea, and adverse COVID-19 outcomes, our findings further support the role of renal metabolic stress as an important prognostic marker in severe illness [19,20].

### Inflammation vs. Impaired physiological homeostasis

The multivariable model results showed that traditional baseline characteristics which included sex and comorbidities were not independently associated after multivariable adjustment, whereas albumin and urea maintained their ability to predict outcomes throughout the study. These findings suggest that the body’s immediate physiological capacity and the patient’s organ function at the time of hospital admission provide better risk assessment than baseline demographic factors after the onset of acute medical conditions.

Inflammatory markers reflect active immune dysregulation, whereas albumin and urea may better represent impaired physiological homeostasis during severe illness. The simultaneous presence of low albumin levels and elevated urea often signals broader systemic instability rather than a problem confined to a single organ. In this setting, the “hepato-renal axis” can be understood as a conceptual framework used to explain the shared functional vulnerability of the liver and kidneys. It does not imply the existence of a proven or mechanistically established biological interaction between the liver and kidneys in the pathophysiology of COVID-19.

These findings are consistent with prior critical care literature suggesting that organ dysfunction markers may provide stronger prognostic discrimination than isolated inflammatory markers.

These findings have significant practical implications. Laboratory tests for albumin and urea are routinely available, reasonably priced, and quickly measurable. These markers are available even in environments with limited resources, unlike interleukin assays or specialized inflammatory panels. In situations where sophisticated biomarkers are not available, incorporating hepatic and renal functional markers into admission triage models may improve early risk stratification. Their potential incorporation into composite clinical risk scores is supported by the strong discrimination seen (AUC 0.86). Discrimination by itself, however, is not equivalent to clinical utility; prior to implementation, prospective validation and calibration assessment would be necessary.

### Strengths and limitations

This study benefits from a relatively large sample size because it uses systematic data validation methods and it handles missing data through multiple imputation. The multivariable framework used in the study combined inflammatory markers with coagulation and metabolic markers to assess their independent prognostic values. The model showed good discrimination ability and it maintained acceptable stability.

Several limitations merit careful consideration. This study was conducted at a single tertiary care center during a defined period of the pandemic, and treatment protocols may have evolved over time. Information on corticosteroid use, antiviral therapies, ICU admission criteria, and vaccination status was not systematically available for analysis. These factors may have influenced both mortality outcomes and laboratory parameters at presentation, and residual confounding related to treatment variability cannot be excluded. Therefore, caution is warranted when generalizing these findings to other settings or to different phases of the pandemic. Moreover, laboratory cut-offs and mortality rates can be affected by hospital guidelines and population parameters.

Second, multiple imputation was performed assuming Missing at Random (MAR), but this assumption cannot be fully verified in retrospective observational datasets. More frequent laboratory testing could have been performed in clinically severe cases of COVID-19 in hospitalized patients, indicating potential informative or non-random missingness. Patients with less severe disease and less abnormal laboratory profiles may have been underrepresented in the analytical dataset, which may have impacted the observed magnitude of association between laboratory markers and mortality. However, sensitivity analyses performed on complete-case data demonstrated findings that were consistent in direction, supporting the robustness of the primary results. Nevertheless, these findings should be interpreted with appropriate caution. Because albumin, urea, LDH, and D-dimer had relatively high proportions of missing data, their estimated associations should be interpreted as exploratory and require confirmation in prospective studies with more complete laboratory ascertainment.

Third, laboratory indicators were only collected on admission; no dynamic changes during hospitalization could be analyzed. Temporal profiles such as declining albumin or rising urea could provide even more prognostic information. Finally, the term “hepato-renal axis” should therefore be interpreted as a conceptual framework rather than a biologically validated pathway. Future mechanistic investigations are necessary to determine whether coordinated organ-level interactions truly underlie the observed associations.

## Conclusion

This study revealed that admission hypoalbuminemia and elevated blood urea were independent predictors of in-hospital mortality in hospitalized adults with COVID-19, even after adjusting for demographic and inflammatory factors. These findings suggest that markers of organ-functional reserve provide prognostic information beyond inflammatory markers. Prospective validation is required before incorporation of these markers into clinical risk-stratification models.

## Supporting information

S1 Table Checklist of the STROBE statement. (DOCX)

S2 Table Comparison of Included and Excluded Patients. (DOCX)

S3 Table Assessment of Normality for Continuous Variable. (DOCX)

S4 Table Complete-Case Sensitivity Analysis for Multivariable Logistic Regression. (DOCX)

S5 Table Multicollinearity Assessment Using Variance Inflation Factors (VIF). (DOCX)

S6 Table Linktest Results for Model Specification. (DOCX)

S7 Table Hosmer–Lemeshow Goodness-of-Fit Test for the Final Multivariable Model. (DOCX)

S8 Table Interaction Analysis Between Serum Albumin and Blood Urea. (DOCX)

S9 Table Supplementary Sensitivity Analysis Including Serum Creatinine in the Multivariable Mortality Model. (DOCX)
