# Supplementary material for: Serum albumin and blood urea as independent predictors of in-hospital mortality in hospitalized COVID-19 patients: A retrospective cohort study
Source: PLoS One. 2026 Jul 8;21(7):e0353456. doi: 10.1371/journal.pone.0353456 (PMC13345233; doi:10.1371/journal.pone.0353456)
Supplement: S1 Table — (DOCX) [file pone.0353456.s001.docx]

**Serum Albumin and Blood Urea as Independent Predictors of In-Hospital Mortality in Hospitalized COVID-19 Patients: A Retrospective Cohort Study**

**S1 Table. Checklist of the STROBE statement**

|  | Item No | Recommendation | Response based on the Manuscript |
| --- | --- | --- | --- |
| **Title and abstract** | 1 | (*a*) Indicate the study’s design with a commonly used term in the title or the abstract | The title and abstract include the study design A Retrospective Cohort Study |
|  |  | (*b*) Provide in the abstract an informative and balanced summary of what was done and what was found | The abstract includes background, methods (design, sample size, analytical techniques), key results (ORs, p-values), and the conclusion. |
| Introduction | | |  |
| Background/rationale | 2 | Explain the scientific background and rationale for the investigation being reported | Yes – The introduction clearly explains the scientific background of COVID-19 mortality, highlights the prognostic role of inflammatory markers, and identifies the knowledge gap regarding hepatic and renal functional markers |
| Objectives | 3 | State specific objectives, including any prespecified hypotheses | Yes - The study objective is explicitly stated: to evaluate the independent and combined effects of serum albumin and blood urea on in-hospital mortality after adjusting for demographic and inflammatory factors. |
| Methods | | |  |
| Study design | 4 | Present key elements of study design early in the paper | Yes- The study is clearly described as a retrospective cohort study early in the Methods section |
| Setting | 5 | Describe the setting, locations, and relevant dates, including periods of recruitment, exposure, follow-up, and data collection | Yes- The setting (tertiary care center in Belgaum, Karnataka, India) and study period (October 2020–September 2021) are clearly specified. |
| Participants | 6 | (*a*) Give the eligibility criteria, and the sources and methods of selection of participants | **Yes-** Eligibility criteria (≥18 years, laboratory-confirmed COVID-19) and exclusion criteria (missing outcome, incomplete demographics, implausible laboratory values) are clearly described. Patient selection and final sample size (n=1,074) are detailed with a flow diagram. |
| Variables | 7 | Clearly define all outcomes, exposures, predictors, potential confounders, and effect modifiers. Give diagnostic criteria, if applicable | Yes- Outcome (in-hospital mortality), main exposures (albumin and urea), confounders (age, sex, comorbidities, inflammatory markers), and interaction term are clearly defined |
| Data sources/ measurement | 8* | For each variable of interest, give sources of data and details of methods of assessment (measurement). Describe comparability of assessment methods if there is more than one group | Yes- Data were extracted from electronic medical records using standardized forms, with manual validation, quality checks (10% rechecked), and predefined laboratory plausibility ranges. |
| Bias | 9 | Describe any efforts to address potential sources of bias | Yes- Potential bias was addressed through data validation, exclusion of implausible records, multiple imputation for missing data, sensitivity analysis (complete-case), multicollinearity testing, and model calibration assessment |
| Study size | 10 | Explain how the study size was arrived at | Yes- The sample size was determined by the total number of eligible patients admitted during the study period. No a priori sample size calculation was performed due to the retrospective design. |
| Quantitative variables | 11 | Explain how quantitative variables were handled in the analyses. If applicable, describe which groupings were chosen and why | Yes- Continuous variables were analyzed using medians (IQR) and logistic regression. Laboratory values were modeled per clinically meaningful increments (urea per 10 mg/dL; LDH per 100 U/L), and skewed variables were log-transformed before imputation |
| Statistical methods | 12 | (*a*) Describe all statistical methods, including those used to control for confounding | Yes- Univariable and multivariable logistic regression analyses were performed. Confounding was controlled by adjusting for age, sex, comorbidities, and inflammatory markers. Variable selection was based on clinical relevance and p < 0.20 threshold. Adjusted odds ratios with 95% CIs were reported. |
|  |  | (*b*) Describe any methods used to examine subgroups and interactions | Yes- An interaction term (albumin × urea) was included in the multivariable model to assess effect modification. The interaction was not statistically significant. |
|  |  | (*c*) Explain how missing data were addressed | Yes- Missing laboratory data (9.7%–33.9%) were handled using Multiple Imputation by Chained Equations (MICE), generating 30 imputed datasets. Estimates were combined using Rubin’s rules. Sensitivity analyses were conducted |
|  |  | (*d*) If applicable, describe analytical methods taking account of sampling strategy | Not applicable  The study used a retrospective cohort of consecutively admitted hospitalized patients; no complex sampling strategy was applied |
|  |  | (*e*) Describe any sensitivity analyses | Yes- A complete-case analysis was performed and yielded effect estimates consistent with the primary imputed model, supporting robustness of findings |
| Results | | |  |
| Participants | 13* | (a) Report numbers of individuals at each stage of study—eg numbers potentially eligible, examined for eligibility, confirmed eligible, included in the study, completing follow-up, and analysed | Yes- 1,246 patients were identified; 172 were excluded; 1,074 were included in the final analysis |
|  |  | (b) Give reasons for non-participation at each stage | Yes- Reasons for exclusion included missing outcome data, incomplete demographics, age <18 years, and implausible or unverifiable laboratory values |
|  |  | (c) Consider use of a flow diagram | Yes- A flow diagram (Figure 1) illustrates patient selection and final analytical cohort. |
| Descriptive data | 14* | (a) Give characteristics of study participants (eg demographic, clinical, social) and information on exposures and potential confounders | Yes –  Baseline demographic, clinical, and laboratory characteristics are presented in Table 1, stratified by survival status. |
|  |  | (b) Indicate number of participants with missing data for each variable of interest | Yes- The proportion of missing data for laboratory variables (ranging from 9.7% to 33.9%) is reported in the statistical methods section |
| Outcome data | 15* | Report numbers of outcome events or summary measures | Yes- n-hospital mortality occurred in 264 patients (24.5%). Mortality counts are clearly reported in the Results section and tables |
| Main results | 16 | (*a*) Give unadjusted estimates and, if applicable, confounder-adjusted estimates and their precision (eg, 95% confidence interval). Make clear which confounders were adjusted for and why they were included | Yes- Crude odds ratios (Table 2) and adjusted odds ratios (Table 3) with 95% confidence intervals and p-values are clearly reported. The multivariable model adjusted for age, sex, comorbidities, and inflammatory markers based on clinical relevance and statistical criteria |
|  |  | (*b*) Report category boundaries when continuous variables were categorized | Not applicable  Continuous variables were analyzed as continuous measures and rescaled to clinically meaningful increments (e.g., urea per 10 mg/dL, LDH per 100 U/L); no categorization was performed. |
|  |  | (*c*) If relevant, consider translating estimates of relative risk into absolute risk for a meaningful time period | Not applicable  The study used logistic regression and reported odds ratios. Absolute risk translation was not performed, and this was not required for the study objectives. |
| Other analyses | 17 | Report other analyses done—eg analyses of subgroups and interactions, and sensitivity analyses | Yes- Interaction analysis (albumin × urea) was conducted and reported as non-significant. Sensitivity analysis using complete-case data was performed and showed consistent results. Model performance was assessed using ROC curve analysis (AUC = 0.86) |
| Discussion | | |  |
| Key results | 18 | Summarise key results with reference to study objectives | Yes- The discussion clearly summarizes the main findings in relation to the study objective, emphasizing the independent prognostic value of albumin and urea |
| Limitations | 19 | Discuss limitations of the study, taking into account sources of potential bias or imprecision. Discuss both direction and magnitude of any potential bias | Yes- Limitations are thoroughly discussed, including single-center design, retrospective nature, missing data assumptions, lack of dynamic laboratory trends, and potential residual confounding |
| Interpretation | 20 | Give a cautious overall interpretation of results considering objectives, limitations, multiplicity of analyses, results from similar studies, and other relevant evidence | Yes- A balanced and cautious interpretation is provided, acknowledging limitations, avoiding causal claims, and comparing findings with existing literature |
| Generalisability | 21 | Discuss the generalisability (external validity) of the study results | Yes- The manuscript discusses limited generalizability due to single-center design and evolving treatment protocols during different pandemic phases |
| Other information | | |  |
| Funding | 22 | Give the source of funding and the role of the funders for the present study and, if applicable, for the original study on which the present article is based | Yes  The study reports that no external funding was received and declares no conflicts of interest. |

*Give information separately for exposed and unexposed groups.

**Note:** An Explanation and Elaboration article discusses each checklist item and gives methodological background and published examples of transparent reporting. The STROBE checklist is best used in conjunction with this article (freely available on the Web sites of PLoS Medicine at http://www.plosmedicine.org/, Annals of Internal Medicine at http://www.annals.org/, and Epidemiology at http://www.epidem.com/). Information on the STROBE Initiative is available at www.strobe-statement.or
