# Supplementary material for: Serum albumin and blood urea as independent predictors of in-hospital mortality in hospitalized COVID-19 patients: A retrospective cohort study
Source: PLoS One. 2026 Jul 8;21(7):e0353456. doi: 10.1371/journal.pone.0353456 (PMC13345233; doi:10.1371/journal.pone.0353456)
Supplement: S2 Table — (DOCX) [file pone.0353456.s002.docx]

**S2 Table. Comparison of Included and Excluded Patients**

| **Variable** | **Included (n=1074)** | **Excluded (n=172)** | **p-value** |
| --- | --- | --- | --- |
| Age Median (IQR) | 54 (25) | 52 (23) | 0.49 |
| Male sex, n (%) | (67.3%) | (62.4%) | 0.13 |
| In-hospital mortality, n (%) | (24.6%) | (20.2%) | 0.07 |
