# Supplementary material for: Serum albumin and blood urea as independent predictors of in-hospital mortality in hospitalized COVID-19 patients: A retrospective cohort study
Source: PLoS One. 2026 Jul 8;21(7):e0353456. doi: 10.1371/journal.pone.0353456 (PMC13345233; doi:10.1371/journal.pone.0353456)
Supplement: S5 Table — (DOCX) [file pone.0353456.s005.docx]

**S5 Table. Multicollinearity Assessment Using Variance Inflation Factors (VIF)**
