# Supplementary material for: Serum albumin and blood urea as independent predictors of in-hospital mortality in hospitalized COVID-19 patients: A retrospective cohort study
Source: PLoS One. 2026 Jul 8;21(7):e0353456. doi: 10.1371/journal.pone.0353456 (PMC13345233; doi:10.1371/journal.pone.0353456)
Supplement: S6 Table — (DOCX) [file pone.0353456.s006.docx]

### ****S6 Table. Linktest Results for Model Specification****
