# Supplementary material for: Serum albumin and blood urea as independent predictors of in-hospital mortality in hospitalized COVID-19 patients: A retrospective cohort study
Source: PLoS One. 2026 Jul 8;21(7):e0353456. doi: 10.1371/journal.pone.0353456 (PMC13345233; doi:10.1371/journal.pone.0353456)
Supplement: S7 Table — (DOCX) [file pone.0353456.s007.docx]

**S7 Table. Hosmer–Lemeshow Goodness-of-Fit Test for the Final Multivariable Model**

**Hosmer–Lemeshow goodness-of-fit**

Goodness-of-fit test after logistic model

Variable: COVID_9_oucome
