# Supplementary material for: Serum albumin and blood urea as independent predictors of in-hospital mortality in hospitalized COVID-19 patients: A retrospective cohort study
Source: PLoS One. 2026 Jul 8;21(7):e0353456. doi: 10.1371/journal.pone.0353456 (PMC13345233; doi:10.1371/journal.pone.0353456)
Supplement: S9 Table — (DOCX) [file pone.0353456.s009.docx]

**S9 Table.** Supplementary Multivariable Logistic Regression Analysis Including Serum Creatinine

Logistic regression Number of obs = 853

LR chi2(17) = 296.58

Prob > chi2 = 0.0000

Log likelihood = -331.10793 Pseudo R2 = 0.3093

--------------------------------------------------------------------------------

COVID_9_oucome | Odds ratio Std. err. z P>|z| [95% conf. interval]

---------------+----------------------------------------------------------------

Age | 1.024186 .0068207 3.59 0.000 1.010904 1.037642

Sex | 1.25445 .295258 0.96 0.335 .790874 1.989754

Comorbidity | 1.015096 .2182617 0.07 0.944 .6660168 1.547137

Hb | 1.044875 .0548768 0.84 0.403 .9426695 1.158163

WBC | 1.064908 .0189883 3.53 0.000 1.028335 1.102783

Platelets | .9982922 .0011647 -1.46 0.143 .9960121 1.000578

NLR | 1.029317 .0105818 2.81 0.005 1.008785 1.050268

LDH | 1.001627 .0003158 5.16 0.000 1.001009 1.002247

hsCRP | 1.003714 .0008818 4.22 0.000 1.001987 1.005444

D_Dimer | 1.000047 .0000231 2.04 0.042 1.000002 1.000092

Urea10 | 1.130794 .0372553 3.73 0.000 1.060083 1.206222

Albumin | .5088272 .0983698 -3.49 0.000 .3483457 .7432418

PT | 1.023409 .014077 1.68 0.093 .9961871 1.051375

Na | 1.017351 .0162298 1.08 0.281 .9860329 1.049663

K | .9268332 .1272794 -0.55 0.580 .7081225 1.213095

HCO3 | .9873427 .0129684 -0.97 0.332 .9622495 1.01309

CERT | .9449931 .0699599 -0.76 0.445 .817358 1.092559

_cons | .0036737 .009149 -2.25 0.024 .0000279 .4840989

--------------------------------------------------------------------------------

Note: _cons estimates baseline odds.

.
