# Supplementary material for: Serum albumin and blood urea as independent predictors of in-hospital mortality in hospitalized COVID-19 patients: A retrospective cohort study
Source: PLoS One. 2026 Jul 8;21(7):e0353456. doi: 10.1371/journal.pone.0353456 (PMC13345233; doi:10.1371/journal.pone.0353456)
Supplement: Attachment — Submitted filename: Point-by-point response to the comments.pdf [file pone.0353456.s011.pdf]

<u>Point-by-point response</u>

Dear Editors and Reviewers,

We would like to sincerely thank you for the time and effort devoted to reviewing our manuscript entitled "**Serum Albumin and Blood Urea as Independent Predictors of In-Hospital Mortality in Hospitalized COVID-19 Patients: A Retrospective Cohort Study**" (Submission ID: PONE-D-26-10160).

We are grateful for the insightful feedback and constructive suggestions, which have helped improve the clarity, methodological rigor, and overall quality of the manuscript and provided us with the opportunity to further strengthen our work.

In response to the Editor's and Reviewers' comments, we have carefully revised the manuscript. Major revisions included substantial condensation of the Introduction, expanded discussion regarding missing data and informative missingness, clarification of the clinical interpretation of albumin findings, refinement of the conceptual framing of the "hepato-renal axis," and inclusion of a supplementary sensitivity analysis incorporating serum creatinine into the multivariable model.

All comments have been carefully addressed and are summarized in the detailed point-by-point response below, with each comment accompanied by the corresponding revision. For ease of review, all changes in the revised manuscript have been highlighted using track changes.

We believe that these revisions have substantially improved the clarity, consistency, methodological transparency, and overall scientific quality of the manuscript. We sincerely appreciate the valuable comments and the opportunity to revise our work, and we hope that the revised version is now suitable for publication in PLOS ONE.

Thank you again for your valuable time and consideration.

<table>
<tr><th colspan="3">Point-by-point response</th></tr>
<tr><th colspan="2">Reviewers Comments</th><th>Author’s revision</th></tr>
<tr><th colspan="3">Reviewer 1</th></tr>
<tr><td>1.</td><td>Introduction length
The Introduction is excessively long. It would benefit from substantial condensation. The key background, the prognostic gap between inflammatory markers and organ-functional markers, and the rationale for examining albumin and urea jointly, can be conveyed more concisely. Several paragraphs repeat ideas already established in earlier sentences. I recommend trimming the Introduction to focus only on what is strictly necessary to motivate the research question.</td><td>Thank you for this valuable suggestion.

The Introduction has been substantially revised and condensed to improve clarity and focus. Repetitive background information and overly detailed pathophysiological explanations were removed, and the section was streamlined to emphasize only the key rationale for the study. Specifically, we shortened the general COVID-19 background, consolidated the discussion of established inflammatory markers, reduced redundant explanations regarding hepatic and renal dysfunction, and simplified the description of the proposed “hepato-renal axis” conceptual framework. The revised Introduction now focuses more directly on the study rationale and research objective.

Introduction Section

Pages 4-5</td></tr>
<tr><td>2.</td><td>Missing data and selection bias
This is my principal methodological concern. The authors report substantial proportions of missing laboratory data: albumin 28.2%, urea 25.0%, LDH 32.8%, and D-dimer 26.3%. Missing data of this magnitude, particularly for the two primary exposure variables, is not simply a statistical inconvenience, it carries a directional bias risk that the manuscript does not adequately address in its primary presentation.</td><td>Thank you for this important methodological observation.

We agree that the degree of missingness in several laboratory variables, particularly albumin, urea, LDH, and D-dimer, introduces the possibility of informative missingness and potential selection bias.

In response, we strengthened both the Methods and Limitations sections to more explicitly acknowledge that the Missing at Random (MAR) assumption cannot be fully verified in retrospective hospital-based datasets.

We also added discussion regarding the possibility that laboratory testing was more frequently performed in clinically</td></tr>
</table>

		severe patients, which may have influenced the observed associations.<br><br>Additionally, we clarified the rationale for retaining variables with substantial missingness in the primary multivariable model, emphasizing their established clinical relevance and the consistency observed in complete-case sensitivity analyses. Finally, we expanded the limitations discussion to encourage cautious interpretation and noted the need for prospective validation in datasets with more complete laboratory ascertainment.<br><br>**Method section**<br><br>Page 8 line 163-166<br><br>**Limitation Section**<br><br>Page 18-19 line (382-394)
3.	Clinical significance of the albumin effect<br>While the albumin association reached statistical significance (AOR 0.52, 95% CI 0.37–0.74), the observed difference in median albumin between survivors and non-survivors was 3.6 g/dL versus 3.2 g/dL, a difference of 0.4 g/dL. I would ask the authors to address the clinical significance of this difference more explicitly. In everyday clinical practice, albumin values of 3.2 g/dL and 3.5 g/dL are not generally treated as meaningfully distinct; both fall within a range that most clinicians would characterize as mild-to-moderate hypoalbuminemia. The authors should discuss whether this magnitude of difference is clinically actionable, and whether the statistical association translates into a decision-relevant threshold. Providing a clinically anchored interpretation, for example, framing the AOR in terms of a 0.5 or 1.0 g/dL increment rather than the per-unit estimate, would strengthen the discussion considerably.	Thank you for this important clinical perspective.<br>We agree that, although the association between albumin and mortality was statistically significant, the absolute difference in albumin levels between survivors and non-survivors was relatively modest and may not represent a clinically actionable threshold in isolation.<br>In response, we revised the Discussion section to provide a more clinically grounded interpretation of the findings and clarified that albumin should be interpreted as a marker of broader physiological vulnerability rather than as a standalone decision-making threshold.<br>We also moderated several statements regarding the strength of the association and emphasized the importance of interpreting albumin in conjunction with other inflammatory and organ-functional markers.<br><br>**Abstract**<br>Page 3, line (48-50)<br>**Result**<br>Page 11, line (233-236)<br>**Discussion Section**<br>Page 15, line 309-316

4.	**Rationale for urea over creatinine** The authors select blood urea as the marker of renal metabolic stress rather than serum creatinine, which is the more widely used and internationally standardized marker of glomerular filtration and renal function. No justification for this choice is provided in the Methods section. I ask the authors to explicitly address this: Was creatinine unavailable or subject to higher rates of missingness in this dataset? Is there a physiological rationale for preferring urea in the context of COVID-19 specifically, for example, its additional sensitivity to catabolic states and protein catabolism, which may be relevant in systemic illness? If creatinine data are available, a comparison of both markers in the multivariable model, or at minimum a supplementary analysis including creatinine, would considerably strengthen the manuscript's conclusions about renal involvement.	Thank you for this insightful suggestion. Serum creatinine data were available for a subset of patients in our cohort. In response to the reviewer's recommendation, we performed an additional supplementary sensitivity analysis including serum creatinine in the multivariable model. Blood urea remained independently associated with mortality after adjustment for creatinine and other covariates, whereas creatinine itself was not independently associated with mortality.<br><br>We have now clarified the rationale for selecting urea in the Methods and Discussion sections. Specifically, urea may reflect not only renal dysfunction, but also systemic catabolic stress, hemodynamic instability, and altered protein metabolism during severe acute illness, which may explain its prognostic relevance in severe COVID-19.<br><br>**Methods section**<br><br>Page 8, line 176-179<br><br>**Result section**<br><br>Page 12, line 247-252<br><br>**Discussion section**<br><br>Page 16, line 332-336
**Reviewer 2**		
	We sincerely thank Reviewer #2 for the positive evaluation of our manuscript and for the constructive feedback provided. The manuscript has been carefully revised and language-polished accordingly	
