# Supplementary material for: Serum albumin and blood urea as independent predictors of in-hospital mortality in hospitalized COVID-19 patients: A retrospective cohort study
Source: PLoS One. 2026 Jul 8;21(7):e0353456. doi: 10.1371/journal.pone.0353456 (PMC13345233; doi:10.1371/journal.pone.0353456)
Supplement: Peer review history

# Peer review: Decision Letter 0

**DOI:** 10.1371/journal.pone.0353456.r001

**Authors:** Siddharth Gosavi

4 May 2026

PONE-D-26-10160

Serum Albumin and Blood Urea as Independent Predictors of In-Hospital Mortality in Hospitalized COVID-19 Patients: A Retrospective Cohort Study

PLOS One

Dear Dr. Al-aghbari,

Thank you for submitting your manuscript to PLOS ONE. After careful consideration, we feel that it has merit but does not fully meet PLOS ONE’s publication criteria as it currently stands. Therefore, we invite you to submit a revised version of the manuscript that addresses the points raised during the review process.

Please submit your revised manuscript by Jun 18 2026 11:59PM. If you will need more time than this to complete your revisions, please reply to this message or contact the journal office at plosone@plos.org. When you're ready to submit your revision, log on to https://www.editorialmanager.com/pone/ and select the 'Submissions Needing Revision' folder to locate your manuscript file.

Please include the following items when submitting your revised manuscript:

- A letter that responds to each point raised by the academic editor and reviewer(s). You should upload this letter as a separate file labeled 'Response to Reviewers'.
- A marked-up copy of your manuscript that highlights changes made to the original version. You should upload this as a separate file labeled 'Revised Manuscript with Track Changes'.
- An unmarked version of your revised paper without tracked changes. You should upload this as a separate file labeled 'Manuscript'.

If you would like to make changes to your financial disclosure, please include your updated statement in your cover letter. Guidelines for resubmitting your figure files are available below the reviewer comments at the end of this letter.

If applicable, we recommend that you deposit your laboratory protocols in protocols.io to enhance the reproducibility of your results. Protocols.io assigns your protocol its own identifier (DOI) so that it can be cited independently in the future. For instructions see: https://journals.plos.org/plosone/s/submission-guidelines#loc-laboratory-protocols. Additionally, PLOS ONE offers an option for publishing peer-reviewed Lab Protocol articles, which describe protocols hosted on protocols.io. Read more information on sharing protocols at https://plos.org/protocols?utm_medium=editorial-email&utm_source=authorletters&utm_campaign=protocols.

As the corresponding author, your ORCID iD is verified in the submission system and will appear in the published article. PLOS supports the use of ORCID, and we encourage all coauthors to register for an ORCID iD and use it as well. Please encourage your coauthors to verify their ORCID iD within the submission system before final acceptance, as unverified ORCID iDs will not appear in the published article. *Only*  the individual author can complete the verification step; PLOS staff *cannot*  verify ORCID iDs on behalf of authors.

We look forward to receiving your revised manuscript.

Kind regards,

Siddharth Gosavi, MBBS, MD Internal Medicine,DNB Internal Medicine

Academic Editor

PLOS One

Journal Requirements:

When submitting your revision, we need you to address these additional requirements.

1. Please ensure that your manuscript meets PLOS ONE's style requirements, including those for file naming. The PLOS ONE style templates can be found at

https://journals.plos.org/plosone/s/file?id=wjVg/PLOSOne_formatting_sample_main_body.pdf and

https://journals.plos.org/plosone/s/file?id=ba62/PLOSOne_formatting_sample_title_authors_affiliations.pdf

2. For studies involving third-party data, we encourage authors to share any data specific to their analyses that they can legally distribute. PLOS recognizes, however, that authors may be using third-party data they do not have the rights to share. When third-party data cannot be publicly shared, authors must provide all information necessary for interested researchers to apply to gain access to the data. (https://journals.plos.org/plosone/s/data-availability#loc-acceptable-data-access-restrictions)

For any third-party data that the authors cannot legally distribute, they should include the following information in their Data Availability Statement upon submission:

1) A description of the data set and the third-party source

2) If applicable, verification of permission to use the data set

3) Confirmation of whether the authors received any special privileges in accessing the data that other researchers would not have

4) All necessary contact information others would need to apply to gain access to the data

3. If the reviewer comments include a recommendation to cite specific previously published works, please review and evaluate these publications to determine whether they are relevant and should be cited. There is no requirement to cite these works unless the editor has indicated otherwise.

Additional Editor Comments:

I have several substantive concerns that I believe should be addressed before the manuscript can be considered for publication.

1. Introduction length

The Introduction is excessively long. It would benefit from substantial condensation. The key background, the prognostic gap between inflammatory markers and organ-functional markers, and the rationale for examining albumin and urea jointly, can be conveyed more concisely. Several paragraphs repeat ideas already established in earlier sentences. I recommend trimming the Introduction to focus only on what is strictly necessary to motivate the research question.

2. Missing data and selection bias

This is my principal methodological concern. The authors report substantial proportions of missing laboratory data: albumin 28.2%, urea 25.0%, LDH 32.8%, and D-dimer 26.3%. Missing data of this magnitude, particularly for the two primary exposure variables, is not simply a statistical inconvenience, it carries a directional bias risk that the manuscript does not adequately address in its primary presentation.

In observational hospital cohorts, sicker patients are systematically more likely to have laboratory tests ordered and completed. The converse, that less severely ill patients are less likely to have these values measured, is equally true. This creates a non-random missingness pattern that may be informative: the very patients with less extreme albumin or urea values may be disproportionately absent from the analytical dataset, potentially inflating the observed associations between these markers and mortality.

The authors appropriately conducted a complete-case sensitivity analysis and found directionally consistent results, which is reassuring. However, the way the analysis is currently structured presents the multiply-imputed model as the primary result, with the complete-case analysis as supplementary. Given the degree of missingness and the specific bias mechanism described above, I would recommend that the authors: (a) provide a more explicit discussion of the informative missingness hypothesis in the Limitations section, acknowledging that the Missing at Random assumption cannot be fully verified in this context; and (b) consider whether variables with >20% missingness should be included in the primary model at all, or whether they should be relegated to secondary analyses with appropriate caveats about their interpretability.

3. Clinical significance of the albumin effect

While the albumin association reached statistical significance (AOR 0.52, 95% CI 0.37–0.74), the observed difference in median albumin between survivors and non-survivors was 3.6 g/dL versus 3.2 g/dL, a difference of 0.4 g/dL. I would ask the authors to address the clinical significance of this difference more explicitly. In everyday clinical practice, albumin values of 3.2 g/dL and 3.5 g/dL are not generally treated as meaningfully distinct; both fall within a range that most clinicians would characterize as mild-to-moderate hypoalbuminemia. The authors should discuss whether this magnitude of difference is clinically actionable, and whether the statistical association translates into a decision-relevant threshold. Providing a clinically anchored interpretation, for example, framing the AOR in terms of a 0.5 or 1.0 g/dL increment rather than the per-unit estimate, would strengthen the discussion considerably.

4. Rationale for urea over creatinine

The authors select blood urea as the marker of renal metabolic stress rather than serum creatinine, which is the more widely used and internationally standardized marker of glomerular filtration and renal function. No justification for this choice is provided in the Methods section. I ask the authors to explicitly address this: Was creatinine unavailable or subject to higher rates of missingness in this dataset? Is there a physiological rationale for preferring urea in the context of COVID-19 specifically, for example, its additional sensitivity to catabolic states and protein catabolism, which may be relevant in systemic illness? If creatinine data are available, a comparison of both markers in the multivariable model, or at minimum a supplementary analysis including creatinine, would considerably strengthen the manuscript's conclusions about renal involvement.

[Note: HTML markup is below. Please do not edit.]

Reviewers' comments:

Reviewer's Responses to Questions

**Comments to the Author**

1. Is the manuscript technically sound, and do the data support the conclusions?

The manuscript must describe a technically sound piece of scientific research with data that supports the conclusions. Experiments must have been conducted rigorously, with appropriate controls, replication, and sample sizes. The conclusions must be drawn appropriately based on the data presented.

Reviewer #1: Yes

Reviewer #2: Yes

**********

2. Has the statistical analysis been performed appropriately and rigorously?

Reviewer #1: Yes

Reviewer #2: I Don't Know

**********

3. Have the authors made all data underlying the findings in their manuscript fully available?

The PLOS Data policy requires authors to make all data underlying the findings described in their manuscript fully available without restriction, with rare exception (please refer to the Data Availability Statement in the manuscript PDF file). The data should be provided as part of the manuscript or its supporting information, or deposited to a public repository. For example, in addition to summary statistics, the data points behind means, medians and variance measures should be available. If there are restrictions on publicly sharing data—e.g. participant privacy or use of data from a third party—those must be specified.

Reviewer #1: Yes

Reviewer #2: No

**********

4. Is the manuscript presented in an intelligible fashion and written in standard English?

PLOS ONE does not copyedit accepted manuscripts, so the language in submitted articles must be clear, correct, and unambiguous. Any typographical or grammatical errors should be corrected at revision, so please note any specific errors here.

Reviewer #1: Yes

Reviewer #2: Yes

**********

5. Review Comments to the Author

Please use the space provided to explain your answers to the questions above. You may also include additional comments for the author, including concerns about dual publication, research ethics, or publication ethics. (Please upload your review as an attachment if it exceeds 20,000 characters)

Reviewer #1: Thank you for the opportunity to review this manuscript. I have several substantive concerns that I believe should be addressed before the manuscript can be considered for publication.

1. Introduction length

The Introduction is excessively long. It would benefit from substantial condensation. The key background, the prognostic gap between inflammatory markers and organ-functional markers, and the rationale for examining albumin and urea jointly, can be conveyed more concisely. Several paragraphs repeat ideas already established in earlier sentences. I recommend trimming the Introduction to focus only on what is strictly necessary to motivate the research question.

2. Missing data and selection bias

This is my principal methodological concern. The authors report substantial proportions of missing laboratory data: albumin 28.2%, urea 25.0%, LDH 32.8%, and D-dimer 26.3%. Missing data of this magnitude, particularly for the two primary exposure variables, is not simply a statistical inconvenience, it carries a directional bias risk that the manuscript does not adequately address in its primary presentation.

In observational hospital cohorts, sicker patients are systematically more likely to have laboratory tests ordered and completed. The converse, that less severely ill patients are less likely to have these values measured, is equally true. This creates a non-random missingness pattern that may be informative: the very patients with less extreme albumin or urea values may be disproportionately absent from the analytical dataset, potentially inflating the observed associations between these markers and mortality.

The authors appropriately conducted a complete-case sensitivity analysis and found directionally consistent results, which is reassuring. However, the way the analysis is currently structured presents the multiply-imputed model as the primary result, with the complete-case analysis as supplementary. Given the degree of missingness and the specific bias mechanism described above, I would recommend that the authors: (a) provide a more explicit discussion of the informative missingness hypothesis in the Limitations section, acknowledging that the Missing at Random assumption cannot be fully verified in this context; and (b) consider whether variables with >20% missingness should be included in the primary model at all, or whether they should be relegated to secondary analyses with appropriate caveats about their interpretability.

3. Clinical significance of the albumin effect

While the albumin association reached statistical significance (AOR 0.52, 95% CI 0.37–0.74), the observed difference in median albumin between survivors and non-survivors was 3.6 g/dL versus 3.2 g/dL, a difference of 0.4 g/dL. I would ask the authors to address the clinical significance of this difference more explicitly. In everyday clinical practice, albumin values of 3.2 g/dL and 3.5 g/dL are not generally treated as meaningfully distinct; both fall within a range that most clinicians would characterize as mild-to-moderate hypoalbuminemia. The authors should discuss whether this magnitude of difference is clinically actionable, and whether the statistical association translates into a decision-relevant threshold. Providing a clinically anchored interpretation, for example, framing the AOR in terms of a 0.5 or 1.0 g/dL increment rather than the per-unit estimate, would strengthen the discussion considerably.

4. Rationale for urea over creatinine

The authors select blood urea as the marker of renal metabolic stress rather than serum creatinine, which is the more widely used and internationally standardized marker of glomerular filtration and renal function. No justification for this choice is provided in the Methods section. I ask the authors to explicitly address this: Was creatinine unavailable or subject to higher rates of missingness in this dataset? Is there a physiological rationale for preferring urea in the context of COVID-19 specifically, for example, its additional sensitivity to catabolic states and protein catabolism, which may be relevant in systemic illness? If creatinine data are available, a comparison of both markers in the multivariable model, or at minimum a supplementary analysis including creatinine, would considerably strengthen the manuscript's conclusions about renal involvement.

Reviewer #2: Dear Author

many thanks for submitting this intersecting peace of work on role of Urea and albumin levels in determining prognosis of covid -19. Please see my comments added in highlighted text.

Although this is a retrospective data set and does not provide any new information, never the less the data is good enough to deserve publication after minor review.

Kind regards

**********

6. PLOS authors have the option to publish the peer review history of their article (what does this mean?). If published, this will include your full peer review and any attached files.

If you choose “no”, your identity will remain anonymous but your review may still be made public.

**Do you want your identity to be public for this peer review?**  For information about this choice, including consent withdrawal, please see our Privacy Policy.

Reviewer #1: **Yes:**  David Rene Rodríguez Lima

Reviewer #2: No

**********

[NOTE: If reviewer comments were submitted as an attachment file, they will be attached to this email and accessible via the submission site. Please log into your account, locate the manuscript record, and check for the action link "View Attachments". If this link does not appear, there are no attachment files.]

To ensure your figures meet our technical requirements, please review our figure guidelines: https://journals.plos.org/plosone/s/figures

You may also use PLOS’s free figure tool, NAAS, to help you prepare publication quality figures: https://journals.plos.org/plosone/s/figures#loc-tools-for-figure-preparation.

NAAS will assess whether your figures meet our technical requirements by comparing each figure against our figure specifications.

# Author comment: Author response to Decision Letter 1

**DOI:** 10.1371/journal.pone.0353456.r002

19 May 2026

Dear Editor and Reviewers,

We sincerely thank you for the careful evaluation of our manuscript and for the constructive comments and suggestions provided. In response to the comments, we have carefully revised the manuscript to improve its clarity, methodological transparency, statistical reporting, and overall scientific quality.

Major revisions include substantial condensation of the Introduction, expanded discussion regarding missing data and informative missingness, clarification of the clinical interpretation of albumin findings, refinement of the conceptual framing of the “hepato-renal axis,” and inclusion of a supplementary sensitivity analysis incorporating serum creatinine into the multivariable model.

All reviewer comments have been addressed in detail in the uploaded point-by-point response document, and all revisions have been highlighted using tracked changes in the revised manuscript.

We appreciate the opportunity to revise our work and respectfully submit the revised manuscript for your consideration.

Attachment Submitted filename: Point-by-point response to the comments.pdf

# Editor report: Decision Letter 1

**DOI:** 10.1371/journal.pone.0353456.r003

**Authors:** Siddharth Gosavi, Siddharth Gosavi

25 Jun 2026

Serum Albumin and Blood Urea as Independent Predictors of In-Hospital Mortality in Hospitalized COVID-19 Patients: A Retrospective Cohort Study

PONE-D-26-10160R1

Dear Dr. Al-aghbari,

We’re pleased to inform you that your manuscript has been judged scientifically suitable for publication and will be formally accepted for publication once it meets all outstanding technical requirements.

Within one week, you’ll receive an e-mail detailing the required amendments. When these have been addressed, you’ll receive a formal acceptance letter and your manuscript will be scheduled for publication.

An invoice will be generated when your article is formally accepted. Please note, if your institution has a publishing partnership with PLOS and your article meets the relevant criteria, all or part of your publication costs will be covered. Please make sure your user information is up-to-date by logging into Editorial Manager at Editorial Manager® and clicking the ‘Update My Information' link at the top of the page. For questions related to billing, please contact billing support.

If your institution or institutions have a press office, please notify them about your upcoming paper to help maximize its impact. If they’ll be preparing press materials, please inform our press team as soon as possible -- no later than 48 hours after receiving the formal acceptance. Your manuscript will remain under strict press embargo until 2 pm Eastern Time on the date of publication. For more information, please contact onepress@plos.org.

Kind regards,

Siddharth Gosavi, MBBS, MD Internal Medicine,DNB Internal Medicine

Academic Editor

PLOS One

Additional Editor Comments (optional):

Thank you for your response.

Reviewers' comments:

# Editor report: Acceptance letter

**DOI:** 10.1371/journal.pone.0353456.r004

**Authors:** Siddharth Gosavi, Siddharth Gosavi

PONE-D-26-10160R1

PLOS One

Dear Dr. Al-aghbari,

I'm pleased to inform you that your manuscript has been deemed suitable for publication in PLOS One. Congratulations! Your manuscript is now being handed over to our production team.

At this stage, our production department will prepare your paper for publication. This includes ensuring the following:

* All references, tables, and figures are properly cited

* All relevant supporting information is included in the manuscript submission,

* There are no issues that prevent the paper from being properly typeset

You will receive further instructions from the production team, including instructions on how to review your proof when it is ready. Please keep in mind that we are working through a large volume of accepted articles, so please give us a few days to review your paper and let you know the next and final steps.

Lastly, if your institution or institutions have a press office, please let them know about your upcoming paper now to help maximize its impact. If they'll be preparing press materials, please inform our press team within the next 48 hours. Your manuscript will remain under strict press embargo until 2 pm Eastern Time on the date of publication. For more information, please contact onepress@plos.org.

You will receive an invoice from PLOS for your publication fee after your manuscript has reached the completed accept phase. If you receive an email requesting payment before acceptance or for any other service, this may be a phishing scheme. Learn how to identify phishing emails and protect your accounts at https://explore.plos.org/phishing.

If we can help with anything else, please email us at customercare@plos.org.

Thank you for submitting your work to PLOS ONE and supporting open access.

Kind regards,

PLOS ONE Editorial Office Staff

on behalf of

Dr. Siddharth Gosavi

Academic Editor

PLOS One
